# Integrated functional networks of process, tissue, and developmental stage specific interactions in *Arabidopsis thaliana*

**DOI:** 10.1186/1752-0509-4-180

**Published:** 2010-12-31

**Authors:** Ana Pop, Curtis Huttenhower, Anjali Iyer-Pascuzzi, Philip N Benfey, Olga G Troyanskaya

**Affiliations:** 1 Computer Science Department, Princeton University, Princeton, NJ, USA; 2 Lewis-Sigler Institute for Integrative Genomics, Princeton University, NJ, USA; 3 Biostatistics Department, Harvard School of Public Health, Boston, MA, USA; 4 Department of Biology and Center for Systems Biology, Duke University, Durham, NC, USA

## Abstract

**Background:**

Recent years have seen an explosion in plant genomics, as the difficulties inherent in sequencing and functionally analyzing these biologically and economically significant organisms have been overcome. *Arabidopsis thaliana*, a versatile model organism, represents an opportunity to evaluate the predictive power of biological network inference for plant functional genomics.

**Results:**

Here, we provide a compendium of functional relationship networks for *Arabidopsis thaliana *leveraging data integration based on over 60 microarray, physical and genetic interaction, and literature curation datasets. These include tissue, biological process, and development stage specific networks, each predicting relationships specific to an individual biological context. These biological networks enable the rapid investigation of uncharacterized genes in specific tissues and developmental stages of interest and summarize a very large collection of *A. thaliana *data for biological examination. We found validation in the literature for many of our predicted networks, including those involved in disease resistance, root hair patterning, and auxin homeostasis.

**Conclusions:**

These context-specific networks demonstrate that highly specific biological hypotheses can be generated for a diversity of individual processes, developmental stages, and plant tissues in *A. thaliana*. All predicted functional networks are available online at http://function.princeton.edu/arathGraphle.

## Background

Plants are complex and diverse organisms and have adapted evolutionarily to almost every ecological niche on the planet. Agricultural and pharmaceutical applications of plant genomics have focused on understanding the metabolic and biochemical potential of specific plant tissues and environmental responses [1]. *Arabidopsis thaliana *is the most common model organism for plants, with a short life cycle, relatively few genes, and a fully sequenced genome [2]. It is a multicellular organism with multiple tissue types and developmental stages, and much of its tissue-specific and stage-specific molecular biology has yet to be determined.

Many *A. thaliana *gene products are functional only in a specific tissue or during a specific developmental period. [3,4]. The ability to predict tissue- or development-stage-specific function from genomic data would aid in appropriately targeting experimental work; doing experiments on every plant structure at each of its development stages individually would be tedious and costly. Additionally, it would be challenging to summarize the resulting genomic data efficiently, since the combinatorics of 30 developmental stages [5] by over 50 plant structures [6] makes a large compendium of predictions unwieldy as raw data. With this as motivation, we have created probabilistic networks providing a data-driven view of protein functional relationships and co-expressions in *A. thaliana*. A functional relationship between two genes indicates that their products are used by the cell to perform a particular biological process (for example, two proteins both participating in the DNA damage response). We assign a probability of interaction between all gene pairs in a specific biological context of interest based on experimental data and expert annotations of such relationships from controlled vocabularies.

Tools like Genevestigator https://www.genevestigator.com, AtGenExpress Visualization Tool http://jsp.weigelworld.org/expviz/expviz.jsp, and ATTED-II http://atted.jp/ enable analysis of expression patterns across microarrays of different types and platforms, but none of these three employ active gene function or functional relationship prediction. In general, each takes a set of genes as input and aggregates raw microarray experimental results into informative plots and tables, for example showing host experiments cluster by plant tissue. ATTED-II also integrates a large collection of microarray experiments and utilizes gene co-expression between gene pairs to suggest genes functionally related to a query. However, they do not provide genes related within specific biological processes, tissues, or developmental contexts. Additional tools such as Genemania http://genemania.org, AraNet http://www.functionalnet.org/aranet/, and STRING http://string-db.org/ do provide data integration for *Arabidopsis thaliana*; however, again, none of these provide tissue, development, or biological context specific inferences. Adding such information improves predictions, as is shown in Additional file 1, in which the inclusion of developmental-specific information consistently improves the accuracy of functional predictions.

We have integrated the abundance of genomic data for *A. thaliana *(over 60 datasets) to construct a compendium of biological networks describing functional relationships and co-expression among *A. thaliana *genes. This compendium demonstrates the usefulness of data integration and includes networks that are "global" in the sense that they describe the overall set of functional interactions predicted to occur among *A. thaliana *proteins, independently of plant tissue, developmental stage, or environmental context [7]. However, most networks in this compendium are context-specific: they describe only the functional relationships predicted to occur at a specific time or in a specific tissue. Context-specific data integration does not use all gold standard genes for training. Rather, it trains and evaluates using a subset of genes present in the biological process, tissue, or development stage of interest. The integration up- or down-weights each integrated dataset on a per-context basis, emphasizing experimental results that are particularly informative in each biological area of interest, and it has been shown to significantly increase predictive accuracy in other organisms [8,9]. In this way, biological researchers can use the system to determine whether a gene or genes of interest behave differently in various development stages or if they are active only in specific parts of the plant.

Here, we investigate over 300 resulting global and context-specific functional networks generated for *A. thaliana *biological processes, tissues, and developmental stages. We evaluated these networks computationally to determine the accuracy of their predictions, and we found that genomic datasets are differentially informative across varied contexts. Gene products' predicted roles and interactions also varied, and we found validation in the literature for specific interactions for many proteins. We highlight several of these interactions for a diversity of developmental and physiological processes, including those for PHOSPHOENYL PYRUVATE/PHOSPHATE TRANSPORTER 2 (AtPPT2) during leaf and root developmental stages, the disease resistance proteins RESISTANCE TO PSEUDOMONAS 1 and 2 (RPS1 and RP2), the root epidermal patterning protein WEREWOLF (WER), and the auxin hormone receptor TRANSPORT INHIBITOR RESPONSE 1 (TIR1). Finally, we provide an intuitive, interactive representation of these results online at http://function.princeton.edu/arathGraphle.

## Results and Discussion

**Figure 1:**
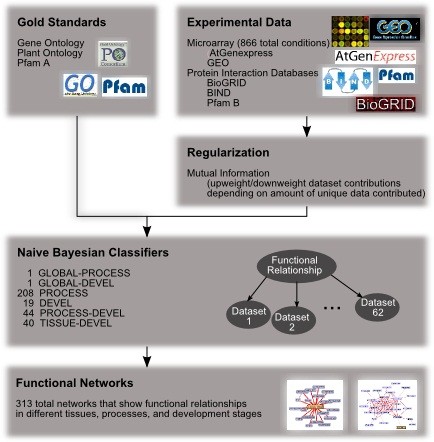
**Schematic of the process, tissue, and developmental stage specific genomic data integration pipeline**. We used regularized Bayesian classifiers [9] to integrate genome-scale data for *A. thaliana *including 55 expression datasets from GEO [38] and 5 physical and genetic interaction datasets from BIND [39] and bioGRID [40]. Using curated biological knowledge from the Gene Ontology [14], Plant Ontology[6], and Pfam [15], we reweighted these datasets to infer genome-wide biological networks focused on individual biological processes, developmental stages, and plant tissues.

We integrated a compendium of *A. thaliana *genomic data (55 microarray and 5 interaction datasets) using a Bayesian framework [10,11] to probabilistic weight each experimental dataset according to its relevance in diverse biological areas (Figure 1). In addition to producing global functional networks summarizing the general interactions occurring among *A. thaliana *genes, we performed additional integrations reweighting the data to emphasize various cellular, developmental, and tissue-specific processes. Each integration is defined by one or more curated gold standards [12], each listing genes whose products are known to be active in the areas of interest (e.g. the photosynthesis pathway, dry seed developmental stage, or leaf tissue). By learning how informative each dataset is with respect to each gold standard, we reweighted the datasets, combined them to infer a single genome-wide functional network in each context of interest, and analyzed the resulting networks as detailed below to generate novel biological hypotheses.

### Overview of integrated functional networks inferred for *A. thaliana *pathways, tissues, and developmental stages

We generated a range of networks (Table 1) addressing questions of increasing specificity regarding *A. thaliana *gene pair relationships. First, this includes two global functional networks representing overall relationships occurring within the *A. thaliana *genome independent of a specific tissue or developmental context. The first, GLOBAL-PROCESS, links genes with high probability if the integrated genomic data indicate that they are employed by the organism in similar biological roles; that is, if they participate in the same cellular processes. The second, GLOBAL-DEVEL, links genes if they are expected to be co-active during the same developmental stage(s).

**Table 1:** Global and context-specific functional relationship networks.

Compendium Type	Compendium Description	Number of Networks	Evaluation (AUC range)
GLOBAL-PROCESS	Global functional network linking genes active in similar biological pathways and processes	1	0.54

GLOBAL-DEVEL	Global functional network linking genes active in the same developmental stage(s)	1	0.63

PROCESS	Networks linking genes active in similar pathways only within the context of each specific biological process	208	0.46 - 0.79

DEVEL	Networks linking genes active in similar developmental stages only within the context of each specific developmental stage	19	0.43 - 0.74

PROCESS-DEVEL	Networks linking genes active in the same pathways during the same developmental stage	40	0.46 - 0.82

TISSUE-DEVEL	Networks linking genes active in the same plant tissues during the same developmental stage	44	0.5 - 0.78

We additionally inferred two compendia of context-specific networks, each describing functional relationships between genes predicted to occur only during a specific biological process or developmental stage. Creating biological process-specific networks (i.e. context-specificity) has been explored for the yeast and human genomes [8,13] and provides a more specific view of genes and their functional interactions tailored to individual biological areas of interest. Here, we expand context-specific inference to include developmental stages and plant tissues in addition to biological processes and pathways. As described in Table 1, this resulted in the PROCESS and DEVEL compendia of networks. Each PROCESS network represents the functional relationships predicted to occur during a specific biological process (e.g. autophagy, the cell cycle, photosynthesis, and so forth), and genes linked with high probability are expected to co-participate in this process. Each DEVEL network represents a plant developmental stage (germination, senescence, etc.), and genes linked with high probability are expected to be co-active in that stage.

Finally, in order to investigate the interactions among biological processes, temporal developmental stages, and spatial locality in tissues, we generated two additional network compendia. The first, PROCESS-DEVEL, includes 40 networks each specific to a process/developmental stage pair (e.g. photosynthesis during leaf senescence). Only 40 of the ~4,000 possible pairs were analyzed due to a lack of curated training data for the remaining process/stage combinations. Similarly, the TISSUE-DEVEL compendium includes 44 networks, each predicting gene pairs expected to be co-active in a specific tissue location and at a specific time during development. All networks in these compendia were inferred using probabilistic Bayesian reweighting of 60 genomic datasets, and the results are analyzed in detail below.

### Context-specific data integration improves predictive accuracy

**Figure 2:**
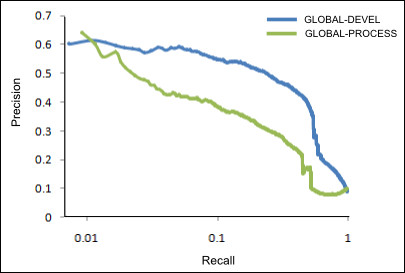
**Performance of the GLOBAL-PROCESS and GLOBAL-DEVEL Networks**. The two global networks were evaluated using 5-fold cross-validation with a 20% holdout gene set to test their ability to accurately recover functional and developmental-stage-specific protein interactions. The higher precision of the GLOBAL-DEVEL network suggests that co-functionality during developmental stages can be more accurately inferred from high-throughput data than can more general functional relationships, although both networks are predicted with significant accuracy.

We evaluated our genome-wide functional network predictions using gold standards based on the Gene Ontology [14], Plant Ontology [6], and Pfam A [15]. This let us determine how accurate each network was in assigning high probability to known functional interactions (i.e. gene pairs co-annotated in GO, PO, etc.) As seen in Figure 2, both the GLOBAL-PROCESS and GLOBAL-DEVEL networks were particularly accurate in the low recall, high precision area of greatest biological interest. Additionally, GLOBAL-DEVEL slightly outperforms GLOBAL-PROCESS, suggesting that gene pairs co-active during the same developmental stages are easier to predict from integrated genomic data than are gene pairs participating in the same biological processes. This is supported intuitively by the fact that developmental expression programs are, in many cases, more sharply defined than are biological pathways and processes, and quantitatively by the fact that several of the integrated datasets explicitly incorporate developmental-stage-specific experiments.

**Figure 3:**
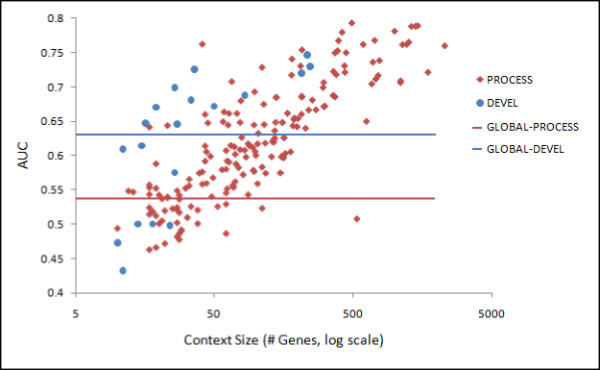
**Context-specific functional networks are often more accurate than global networks**. AUC values for 208 biological process contexts (PROCESS networks) and 19 development contexts (DEVEL networks). The lines indicate the GLOBAL-PROCESS and the GLOBAL-DEVEL networks' performance.

We further found that the context-specific networks usually performed better than the global networks (Figure 3). As the network generation process is data-driven, the accuracy of each integration depends on (1) whether relevant biological signals are present in the data and (2) the availability of a sufficiently comprehensive gold standard. We determine the performance using an AUC (area under the receiver-operator curve) value, which measures the probability that our classifier ranks a functional relationship better than a random classifier. For example, the floral organ development stage context with 34 genes has an AUC of 0.51. Contexts with very limited prior knowledge or a small number of genes annotated to them sometimes perform marginally. Overall more than half (55%) of developmental-stage specific integrations had AUCs over 0.63, that of the GLOBAL-DEVEL network. Many (74%) of the biological process specific integrations had AUCs over 0.54, that of the GLOBAL-PROCESS network. In addition to providing increased predictive power, these context-specific networks focus a very large collection of *A. thaliana *genomic data into individual areas of interest, enabling rapid and directed biological hypothesis generation.

Table 2 details the combinations of developmental stages and tissues/biological processes in the TISSUE-DEVEL and PROCESS-DEVEL compendia for which adequate gold standards were available for evaluation. Networks in plant structures such as embryo and carpel were generally predicted with higher accuracy than those in structures such as leaf and root. AUCs were particularly high in all development contexts and the leaf tissue and were particularly low in all tissues/biological processes for the germination development stage.

**Table 2:** Development stages and tissues/biological processes of interest

Development Stage	Tissue/Biological Process	AUC	Level
C globular stage	meristem	0.822	Strong interaction with development
	leaf	0.818	
	seed	0.754	

D bilateral stage embryo dev stages flower dev stages	meristem	0.816	Strong interaction with development
		0.8	
		0.79	

0 germination flora organ dev stages flower dev stages	carpel	0.66	Weak interaction with development
		0.73	
		0.71	

These nine tissue/process contexts had sufficient overlapping curated information to evaluate our accuracy in predicting functional relationships occurring during a specific developmental stage within one tissue. For example, the meristem activates gene programs to differentiate into shoot and root tissues during the D bilateral stage [20], and we accurately recover these predicted interactions.

The globular stage and meristem combination network has the highest AUC in the TISSUE-DEVEL compendium, and the globular stage is indeed when primary meristems produce new cells that will ultimately differentiate and patterning of the shoot and root apical meristems begins [16]. The globular stage also has a high AUC with other tissues (leaf, root, and seed) and biological processes (the organismal physiological process, the reproductive physiological process, and transcription), suggesting that meristem activity in these tissues is prominent and significant. Other predictions for the meristem [17] are also informative: in the bilateral stage, the meristems become distinguished as shoot and root meristems; in the embryo development stages, the embryo develops radial patterning and primary shoot meristems are formed; and in the flower development stage, floral meristem genes help the transition from shoot to floral meristem [18]. All of these TISSUE-DEVEL networks achieve high AUCs. In contrast, a specialized tissue like the carpel has both low and high predictive powers across development stages. Since the stigma, not carpel, is the receptive tissue where pollen germination happens [19], accuracy is low in the pollen germination development stage but higher in the flower development stage and floral organ development stages.

### Bayesian integration highlights experimental datasets informative in specific biological contexts of interest

**Figure 4:**
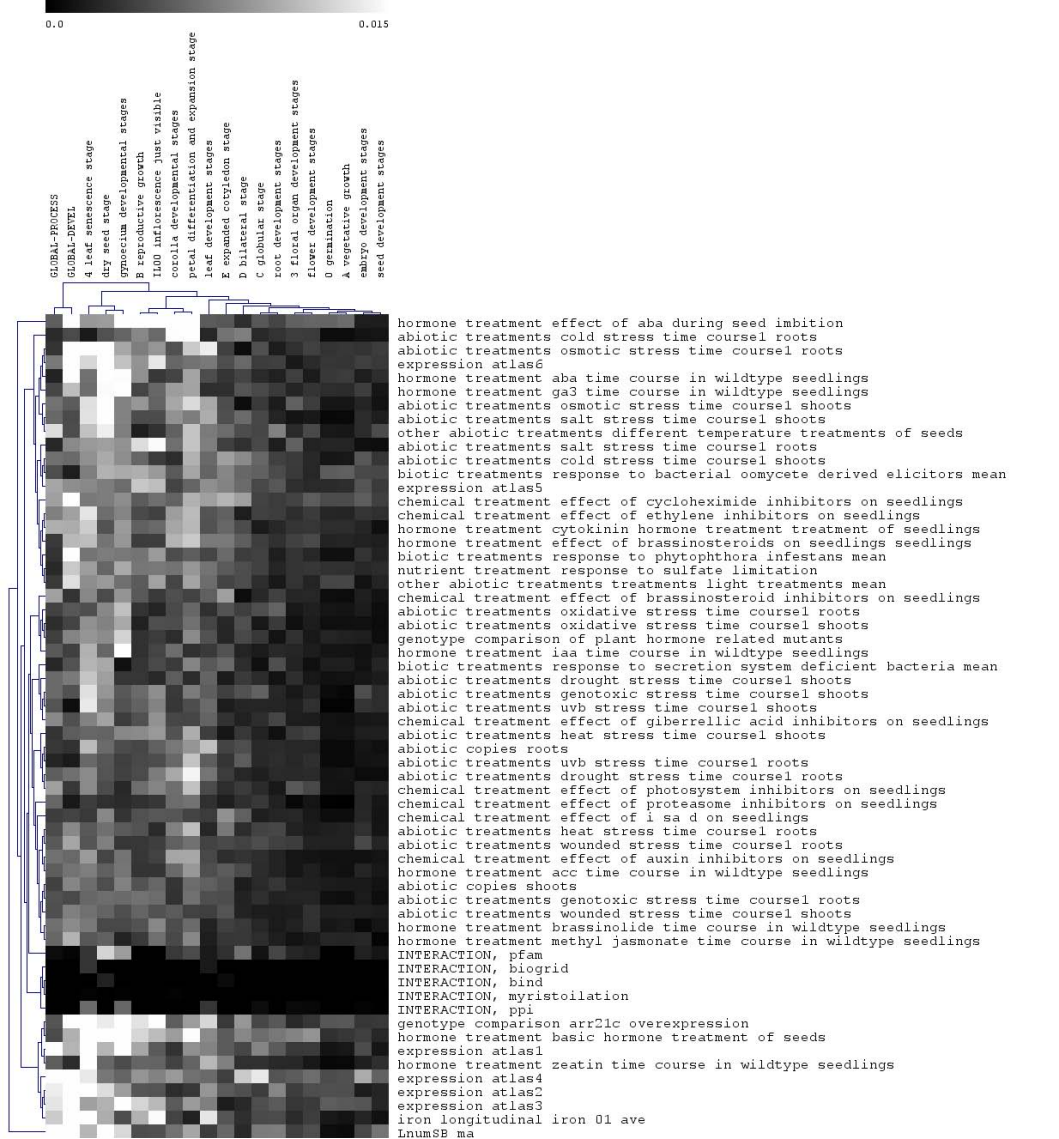
**Weights automatically determined for each dataset contributing to predictions in each context**. Weights are calculated as the influence of each dataset on the posterior probability in the process or development network's Bayesian classifier, where a higher number indicates a greater influence.

We summarize the "weight" given to each dataset during Bayesian integration by calculating its overall influence on the posterior probability of functional relationship. This provides a measure of how informative each dataset is within each context of interest (Figure 4). Highly specific datasets such as physical interactions tend to be informative in many process and developmental contexts. The GLOBAL-PROCESS network, which is the most diffuse and difficult to predict, is not strongly influenced by most datasets and focuses on those that are particularly large and/or diverse. The GLOBAL-DEVEL network, unsurprisingly, is highly influenced by expression datasets incorporating developmental-stage-specific exposures (e.g. hormone treatments and the *A. thaliana *expression atlas [20]). The heterogeneity of dataset contributions increases as context size shrinks, until the smallest contexts are heavily influenced by particularly relevant data (e.g. chemical treatments of seedlings is highly informative in the dry seed stage).

### Regularization of Bayesian network parameters using dataset mutual information efficiently increases prediction accuracy

Naïve Bayesian models assume independence between all input datasets, which can artificially inflate predicted probabilities when this assumption is violated (e.g. when multiple very similar datasets are integrated). Conversely, a full Bayesian model accounting for naturally-occurring dependencies (similar experimental conditions, platform and lab effects, etc.) would be inefficient to learn and evaluate using dozens of whole-genome datasets. Our solution to this issue was to regularize the Bayesian learning process using mutual information between datasets as a prior to upweight or downweight the total possible contribution of each dataset. This mixes a uniform prior with each dataset's predictions, weighted relative to the amount of information it shares with other datasets, and does so as a preprocessing stage without diminishing the efficiency of naive Bayesian learning and inference. We show in Additional file 2 that regularization is critical to the accuracy of our networks (the GLOBAL-PROCESS network substantially outperforms the GLOBAL-PROCESS without regularization; similarly, the GLOBAL-DEVEL network outperforms the GLOBAL-DEVEL without regularization).

Additional file 3 shows normalized pairwise mutual information scores between all datasets. As expected, physical interaction datasets cluster together and are quite different from the main body of microarray expression data. Microarray data falls into several large classes: abiotic stresses, biotic stresses, chemical treatments, hormone treatments, and physical protein-protein interactions. Abiotic treatments are the most similar (and thus downweighted), since they evoke strong transcriptional responses that are easy to detect during the integration process [21-23]. Similarly, other abiotic treatments - different temperature treatments of seeds and hormone treatment - basic hormone treatment of seeds are similar and share more data than most dataset pairs. These datasets are unique in that they stress *A. thaliana *seeds as opposed to seedlings, and their upweighting (Figure 4) may indicate that the response to these stresses is easier to detect in seeds than in other experimental conditions.

### Development-specific networks enable biological hypothesis generation

**Figure 5:**
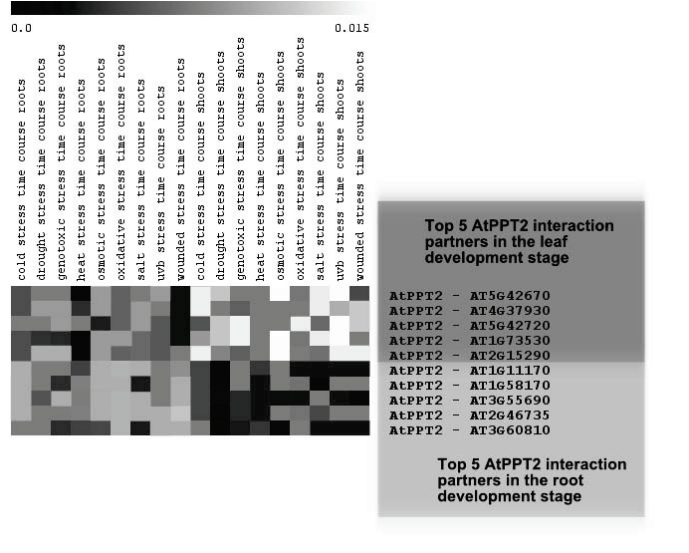
**Information contributed by root and shoot experiments in the leaf and root development contexts**. Predicted interaction partners for AtPPT2 in the leaf and root development stages. In the former case, experiments in shoots are approximately twice as informative as those in roots; the reverse is true in the latter case. This suggests that our network inference process can correctly learn which datasets are most informative in specific contexts.

As an example of biological hypothesis generation using the DEVEL networks, we investigated the most confident interactions predicted for a specific protein, *AtPPT2 *(*AT3G01550*) within two development stages. *AtPPT2 *encodes a PHOSPHOENOLPYRUVATE (PEP)/PHOSPHATE TRANSLOCATOR (PPT) [24] that mediates cytosol-plastid PEP transport [25]. It is highly associated with several genes in the leaf development stage (Additional file 4), but it lacks the same activity in the root development stage. Given this difference, we investigated its top 5 predicted interaction partners in each tissue context. In root development, we found that datasets containing experiments done on the root contributed over 2 times more information (based on posterior probability, Figure 5) than the same experiments done on the shoots. The opposite effect was observed in the leaf context, with experiments on roots downweighted and leaf experiments upweighted. For both root and leaf development, the protein-protein interaction datasets did not have much influence at all compared to the microarray datasets on any of the pairs.

An interesting case study is the predicted functional relationship between genes *AT4G37930 *and *AtPPT2 *in the leaf development stage, which is most influenced by the following datasets: 1) a study of drought stress in shoots [20], 2) salt stress in shoots [20], 3) UVB stress in shoots [20], 4) osmotic stress in shoots [20], and 5) cold stress in shoots [20]. A clear hypothesis implied by this prediction is thus that *AT4G37930 *and *AtPPT2 *both play a role in the cellular response to stress in shoots. Additional experiments not included in our input data [25] show that *AtPPT2 *is highly expressed only in leaf development stages and not in the root development stages.

### Predicted interactions in several networks are literature-validated

RPM1 INTERACTING PROTEIN 4 (RIN4), RESISTANCE TO PSEUDOMONAS SYRINGAE pv. MACULICOLA 1 (RPM1) and RESISTANCE TO PSEUDOMONAS SYRINGAE 2 (RPS2) were predicted to be co-active in the GLOBAL-PROCESS network and in the vegetative growth stages. RIN4 has been shown to physically interact with RPM1 and RPS2, and the three proteins are part of the plant's defense response to the bacterium *P*. *syringae *[26,27]. In the vegetative stage, RIN4 is also predicted to be co-active with NDR1, which physically interacts with RIN4 *in vivo *[28]. Further, in the GLOBAL-DEVEL network, RIN4 is predicted to be co-active with NPR1-like protein 4 (NPR4). Mutations in NPR4 result in susceptibility to *P. syringae*, and although NPR4 has not previously been shown to associate with RIN4, our predicted network suggests these proteins may interact.

Our GLOBAL-DEVEL network predicts an interaction between the root hair patterning regulator WEREWOLF (WER) and additional proteins in the root hair development pathway, including CAPRICE (CPC), GLABRA3 (GL3), and ENHANCER OF GLABRA3 (EGL3). In addition, this network predicts that GL3 and EGL3 interact, and that CPC is interacts with EGL3 and GL3. WER is known to regulate expression of CPC [29], and both WER and CPC regulate expression of EGL3 and GL3 [30]. Further, GL3 and EGL3 physically interact [31]. We also found that the transcription factors (TFs) MAGPIE (MGP), NUTCRACKER (NUC) and JACKDAW (JKD) are co-active in the seedling growth stage, while MGP and NUC are co-active in the root development stages. These three proteins are part of a network involved in ground tissue patterning in the root [32,33]. MGP and NUC are downstream direct targets of the ground tissue patterning regulator SHORTROOT (SHR) [32]. JKD and MGP physically interact both with each other and with SHR and another key ground tissue patterning transcription factor (TF), SCARECROW (SCR) [33]. *MGP *transcription depends on SHR and SCR, while *JKD *transcription in embryogenesis is independent of SHR and SCR, but becomes dependent on these TFs at later stages [33]. Though *mgp *mutants do not have a phenotype, *jkd *mutants show a small reduction in root length compared to wild type plants. Additionally, reducing *MGP *expression in the *jkd *mutant showed that these proteins have opposing effects on SHR and SCR in the ground tissue [33].

A third predicted network involves the plant hormone auxin. *TRANSPORT INHIBITOR RESPONSE 1 *(*TIR1*), encodes an auxin receptor that regulates auxin-mediated transcription [34,35]. TIR1 has been shown to interact with ASK1, ASK2, AtCUL1, and AUX/IAA proteins [36,37], all of which are predicted to be co-active in the GLOBAL-DEVEL network. Our network further predicts that TIR1 interacts with proteins not known to associate with the receptor, such as AT3G23640, a heteroglycan glucosidase involved in carbohydrate metabolism, and AT2G36720, an uncharacterized transcription factor, suggesting that these proteins may be involved in auxin related processes.

Together, these results show that our networks can accurately predict interactions in different plant developmental stages in a wide array of physiological processes.

## Conclusions

Here, we present an ensemble of genome-wide functional relationship networks predicted for *A. thaliana *using Bayesian integration of 60 experimental datasets. ArathGraphle is a hypothesis generation tool that integrates information from a variety of experiments to find consistent co-activities that might otherwise go unnoticed. We infer six classes of networks: one GLOBAL-PROCESS network predicting genes participating in related biological roles; one GLOBAL-DEVEL network predicting genes co-active in the same developmental stage(s); a compendium of PROCESS networks, each containing relationships specific to one biological process or pathway; a compendium of DEVEL networks, each predicting co-activity within an individual developmental stage; and the PROCESS-DEVEL and TISSUE-DEVEL compendia calling out processes and tissue-specific activity occurring during individual developmental stages. Each network reweights the genomic data compendium to yield predictions tailored to an individual biological context of interest. The leaf- and root-specific networks predicted that the AtPPT2 protein functions during leaf development but not root development, which has since been confirmed experimentally [25]. We further identified several literature-validated interactions among our predicted interactions.

We anticipate that these context-specific predictions of *A. thaliana *functional relationships will be useful to drive future hypotheses generation regarding protein function and interactions as they change among *A. thaliana *tissues and developmental stages. With these networks, biologists can pose questions regarding individual genes' interactions within isolated plant tissues and at only one (or more) time(s) during development, allowing them to discover novel functional interactions more rapidly. A web interface to our predictions, available at http://function.princeton.edu/arathGraphle, provides these networks in a convenient interface accessible to the wider biological and bioinformatics communities.

## Methods

The experimental framework for this study consisted of the following processes: three primary gold standards were created indicating genes related or unrelated within biological processes, developmental stages, or plant tissues; *A. thaliana *genomic data was assembled and integrated using regularized Bayesian classifiers; and the resulting predicted genome-wide functional networks were evaluated computationally and experimentally.

### Gold standard generation

We created three gold standards, each containing subsets of positive (related) and negative (unrelated) protein pairs. For the GLOBAL-PROCESS standard, we selected a set of interesting terms from the Gene Ontology as described by [12]. Gene pairs co-annotated to one of these terms were considered to be related, and pairs containing genes annotated to some term (but not co-annotated) were considered to be unrelated. For details, see [11]. This resulted in 188,343 positive and 1,183,813 negative pairs in the GLOBAL-PROCESS standard.

The GLOBAL-DEVEL standard was created similarly, save that genes were required to be co-annotated to a development stage in the Plant Ontology. These gold standards were decomposed into subsets for the PROCESS and DEVEL compendia by limiting positive pairs to individual processes and development stages, respectively, and randomly sub-sampling ten times as many negatives. The PROCESS-DEVEL and TISSUE-DEVEL standards intersected these PROCESS and DEVEL gold standards with an identically generated pathway- and tissue-specific standard using 43 PO terms.

### Bayesian data integration

Each functional relationship network was predicted by a corresponding Bayesian classifier trained as detailed in [11] and [9]. Briefly, a naive classifier was constructed for each gold standard as described above: one each for GLOBAL-PROCESS and GLOBAL-DEVEL, 208 PROCESS terms from the Gene Ontology, 19 DEVEL terms from the Plant Ontology, and 40 PROCESS-DEVEL intersections and 44 TISSUE-DEVEL intersections (each containing at least 10 genes).

Each classifier integrated the same data, broadly comprising coexpression data, protein sequence families, and physical and genetic protein-protein interactions 55 microarray datasets were gathered from AtGenExpress [20] and GEO [38] and converted into pairwise scores by Pearson correlation, z-transformation to obtain a normal distribution $Z=\frac{1}{2}\log\frac{1+p}{1-p}$, and z-scoring to distribute this with mean 0, standard deviation 1 for each dataset. These coexpression scores were discretized into 7 bins from -∞ to -1.5, -1.5 to -0.5, -0.5 to 0.5, 0.5 to 1.5, 1.5 to 2.5, 2.5 to 3.5, 3.5 to ∞. Protein families were drawn from the automatically generated PFam B [15], and protein interactions were taken from BIND [39], BioGRID [40], computational predictions and enzyme assays used for functional annotations [41], and annotations extracted from literature in TAIR (The Arabidopsis Information Resource); all were binarized to indicate the presence or absence of an interaction. This resulted in 60 total datasets integrated in each classifier.

### Regularization using mutual information

Naive Bayesian classifiers assume that all datasets are independent, which becomes increasingly less true as large amounts of biologically similar data are integrated. As detailed in [9], this leads to overconfident and less accurate predictions, which we resolve without loss of efficiency by regularizing the naive classifiers. This process mixes in a uniform prior with weight exponentially proportional to the amount of information shared by each dataset, thus downweighting datasets with less unique information. Mutual information was calculated between each pair of datasets I(*D_k_*; *D_i_*) using the discretization described above and, for each dataset pair, converted to a fraction by dividing by the total amount of possible shared information, I'(*D_k_*; *D_i_*) = I(*D_k_*; *D_i_*)/min(H(*D_k_*), H(*D_i_*)). These fractions were summed for each dataset, $U_{k}=\sum_{i\neq k}I^{'}\left(D_{k};D_{i}\right)$, and exponentially weighted as *α_k _*= 2^*Uk*+1 ^- 1. In combination with Laplace smoothing tune-able with parameter *β_k _*= 2, this yields a regularized classification probability between genes *g_i _*and *g_j_*:

$$P_{i,j}\left(\text{FR}\right)\propto \prod_{k=1}^{n}\frac{\beta_{k}P\left(D_{k}=d_{k}\left(g_{i},g_{j}\right)\right)+\alpha_{k}}{\beta_{k}|D_{k}|+\alpha_{k}|d_{k}|}$$

where *P_i, j_(FR) *is the probability that genes *i *and *j *have a functional relationship, *d_k_(g_i_, g_j_) *is the supporting data for a dataset *k *between a pair of genes *g_i _*and g_*j *_, *P(D_k _= d_k_(g_i_, g_k_)) *is the probability of the dataset *k *containing some value for a pair of genes.

### Computational performance evaluation

We randomly withheld 20% of genes from the positive pairs and 20% from the negative pairs in our gold standard set, using any gene pair including at least one of these genes as a test set excluded during training. All performance evaluations were performed exclusively on test sets selected this way using 5-fold cross validation.

## Authors' contributions

AP performed the computational experiments. AP, CH, and ASI wrote the manuscript. ASI and PNB performed the laboratory experiments. CH and OGT conceived the study, and OGT directed its design and coordination. PNB and OGT helped prepare the final manuscript, which all authors read and approved.

## Supplementary Material

Additional file 1 **Precision-recall plot showing the performance of AraNet versus and GLOBAL-DEVEL**. We show that AraNet does not outperform our GLOBAL-DEVEL network when tested on the developmental gold standards, thus reiterating that adding developmental information improves predictions more than if no developmental information was used. Click here for file

Additional file 2 **Precision-recall plot showing the performance of regularized versus unregularized networks**. To account for possible dependencies between datasets, we used mutual information to regularize the data. We show that the precision-recall plots for the GLOBAL-PROCESS and GLOBAL-DEVEL networks do better than the corresponding networks without having performed regularization. Click here for file

Additional file 3 **Normalized pairwise mutual information scores between all datasets**. To regularize the Bayesian classifiers used in this study, we calculated the mutual information between each pair of datasets. These values were normalized as fractions of the total possible shared information and used to exponentially downweight datasets containing a large fraction of redundant information. The raw mutual information values are shown here and serve to group datasets that are related for technical (e.g. similar microarray platform) or biological (e.g. similar experimental treatment) reasons. Click here for file

Additional file 4 **Functional interactions of AtPPT2 in leaf and root development stages**. To determine whether AtPPT2 was more functionally active in the leaf development stage or the root development stage, we queried the protein AtPPT2 in these two development contexts. We show that the top interactions of this gene are higher in the leaf context than in the root context. Click here for file
