# Supplementary figures and images for: Integrated functional networks of process, tissue, and developmental stage specific interactions in Arabidopsis thaliana
Source: BMC Syst Biol. 2010 Dec 31;4:180. doi: 10.1186/1752-0509-4-180 (PMC3023688; doi:10.1186/1752-0509-4-180)

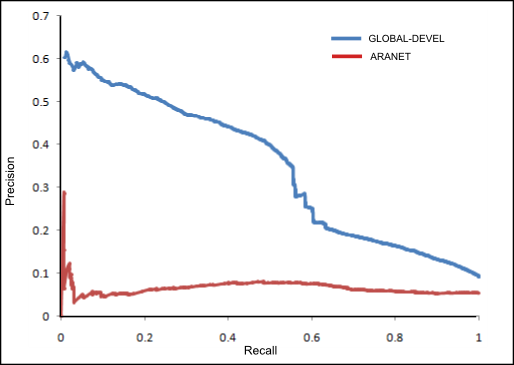

Supplement: Additional file 1 — Precision-recall plot showing the performance of AraNet versus and GLOBAL-DEVEL. We show that AraNet does not outperform our GLOBAL-DEVEL network when tested on the developmental gold standards, thus reiterating that adding developmental information improves predictions more than if no developmental information was used. [file 1752-0509-4-180-S1.PNG]

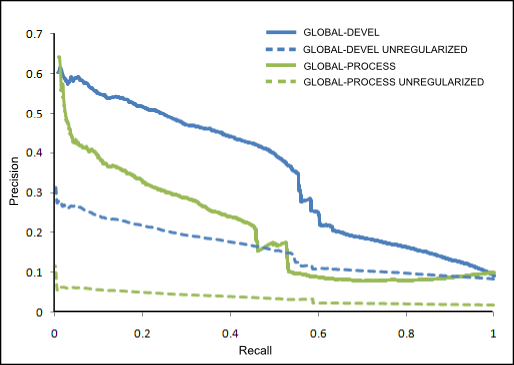

Supplement: Additional file 2 — Precision-recall plot showing the performance of regularized versus unregularized networks. To account for possible dependencies between datasets, we used mutual information to regularize the data. We show that the precision-recall plots for the GLOBAL-PROCESS and GLOBAL-DEVEL networks do better than the corresponding networks without having performed regularization. [file 1752-0509-4-180-S2.PNG]

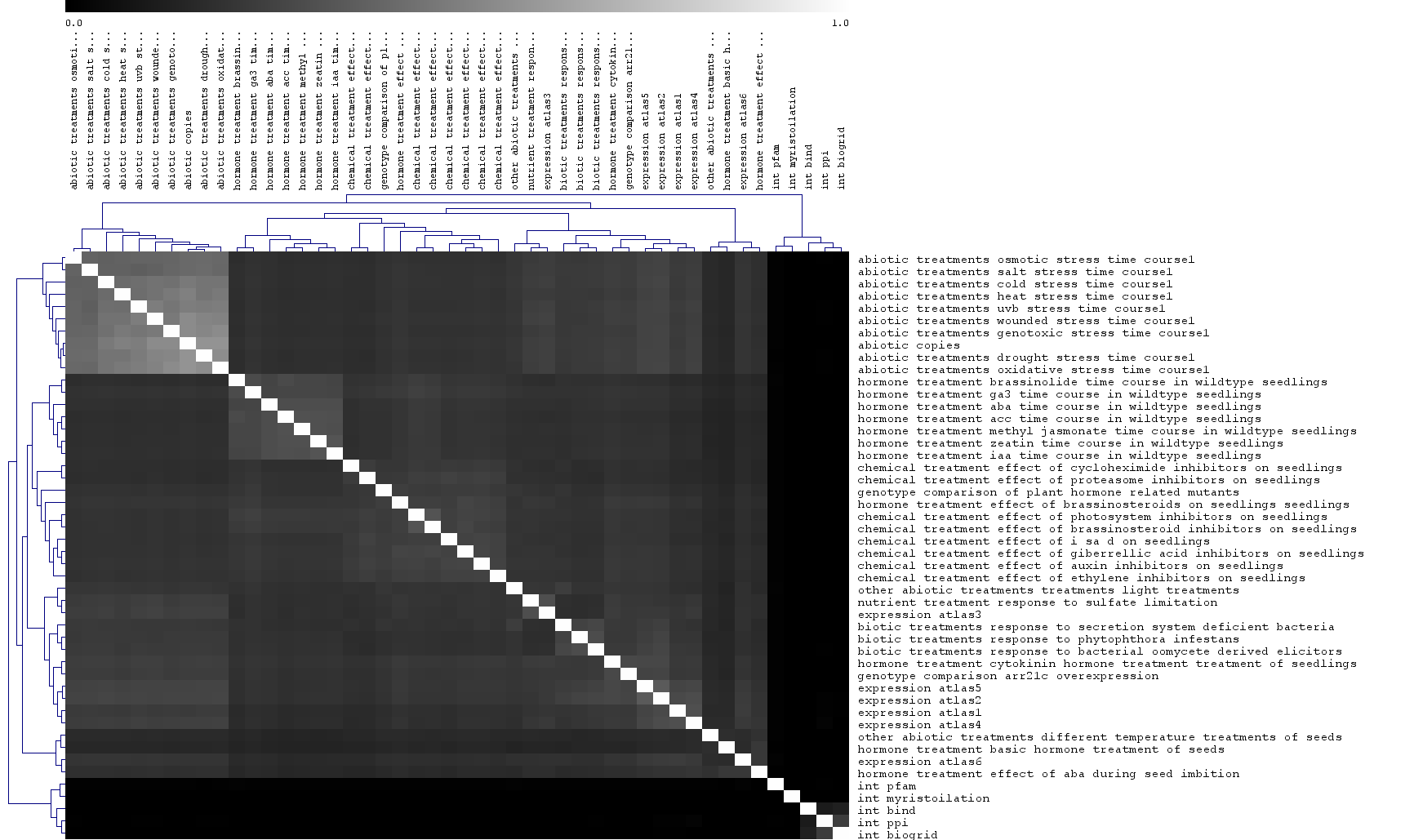

Supplement: Additional file 3 — Normalized pairwise mutual information scores between all datasets. To regularize the Bayesian classifiers used in this study, we calculated the mutual information between each pair of datasets. These values were normalized as fractions of the total possible shared information and used to exponentially downweight datasets containing a large fraction of redundant information. The raw mutual information values are shown here and serve to group datasets that are related for technical (e.g. similar microarray platform) or biological (e.g. similar experimental treatment) reasons. [file 1752-0509-4-180-S3.PNG]
